# From fang to lung: pulmonary embolism following snake envenomation (a case report)

**DOI:** 10.11604/pamj.2026.53.15.50369

**Published:** 2026-01-14

**Authors:** Oussama Mhanni, Mourad Hmidouch, Soumia Boulouiz, Zakaria Bazid, Nabila Ismaili, Noha El Ouafi

**Affiliations:** 1 Laboratory of Epidemiology, Clinical Research and Public Health (LERCSP), Faculty of Medicine and Pharmacy, Mohammed Ist University, Oujda, Morocco,; 2 Department of Cardiology, Mohammed VI University Hospital, Mohammed Ist University, Oujda, Morocco

**Keywords:** Snake bite, envenomation, pulmonary embolism, thrombosis, anticoagulation, case report

## Abstract

Pulmonary embolism is an uncommon complication of snake envenomation. While venom-induced consumption coagulopathy typically produces hemorrhagic manifestations, thrombotic complications can paradoxically develop, requiring careful timing of anticoagulation given competing hemorrhagic risks. We report a 65-year-old woman without thromboembolic risk factors who presented 3 days after a snake bite with progressive lower limb swelling and dyspnea. Physical examination revealed unilateral edema with visible fang marks. Despite normalized coagulation parameters, markedly elevated D-dimers prompted computed tomography pulmonary angiography, which demonstrated bilateral segmental pulmonary embolism without detectable lower extremity deep vein thrombosis. The patient was treated with therapeutic anticoagulation using low-molecular-weight heparin followed by rivaroxaban for 6 months, achieving complete resolution without hemorrhagic complications. Snake venom disrupts hemostasis through a biphasic response: initial consumption coagulopathy depleting factors and platelets, potentially followed by a compensatory prothrombotic phase with hyperfibrinogenemia and thrombocytosis. Additionally, specific venom proteases directly cleave fibrinopeptides A and B from fibrinogen while activating factor XIII, creating stable cross-linked fibrin deposits resistant to fibrinolysis. The absence of deep vein thrombosis suggests thrombogenesis occurs through in situ pulmonary mechanisms or undetectable microemboli. The favorable outcome supports safe initiation of therapeutic anticoagulation once acute coagulopathy resolves. Pulmonary embolism, though rare, can complicate snake envenomation even when coagulation parameters are normalized and deep vein thrombosis is absent. After coagulopathy resolves, anticoagulation is safe. Monitoring for thromboembolic events beyond the acute hemorrhagic phase remains essential.

## Introduction

Pulmonary embolism represents an uncommon complication of snake envenomation, with limited documentation in published literature. While venom-induced consumption coagulopathy typically produces hemorrhagic manifestations, thrombotic complications may develop beyond the acute phase through mechanisms that remain incompletely understood [1]. The optimal timing for therapeutic anticoagulation in this context requires careful consideration given the competing risks of ongoing coagulopathy and thromboembolic progression. We report the case of a 65-year-old woman who developed bilateral segmental pulmonary embolism three days after a snake bite, presenting without conventional thromboembolic risk factors. This case discusses the pathophysiological mechanisms, diagnostic approach, and therapeutic management of this rare complication.

## Patient and observation

**Patient information:** we report the case of a 65-year-old female patient who presented to the emergency department 3 days after sustaining a snake bite to her lower limb, due to progressive swelling and dyspnea. The patient had no significant past medical history, no cardiovascular risk factors, and was not on any chronic medications. There was no personal or family history of thromboembolic events or thrombophilia. She had no history of recent surgery, immobilization, or long-distance travel.

**Timeline of current episode:** the patient sustained a snake bite to her lower limb on day 0. Over the following days, she developed progressive swelling of the affected limb. On day 3 post-bite, she presented to the emergency department due to worsening swelling and the onset of dyspnea.

**Clinical findings:** on admission, the patient was conscious, hemodynamically stable with a blood pressure of 110/60 mmHg and a heart rate of 80 beats per minute. She was afebrile. However, she presented with significant respiratory distress, with oxygen saturation of 88% on room air and respiratory rate of 32 breaths/minute. Physical examination revealed visible fang marks on the lower limb, with marked edema extending from the foot to the mid-thigh, but no signs of compartment syndrome or necrosis. There were no signs of systemic bleeding or neurological involvement. Cardiopulmonary auscultation was unremarkable, and there were no clinical signs of right heart failure.

**Figure 1:**
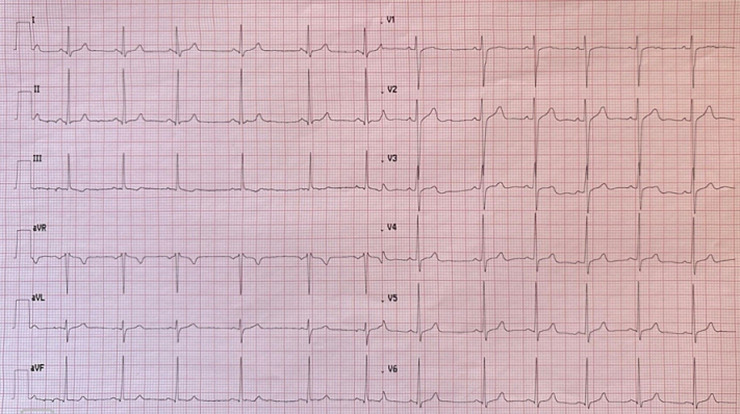
electrocardiogram on admission showing normal sinus rhythm without signs of right ventricular strain

**Figure 2:**
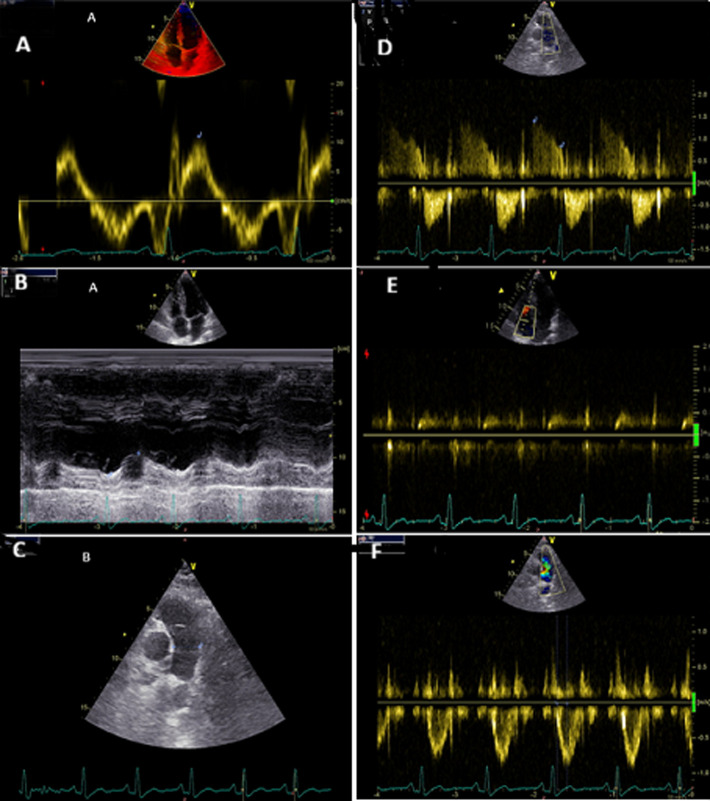
transthoracic echocardiography demonstrating normal right ventricular function without signs of pulmonary hypertension or right heart strain; A) right ventricular systolic function assessment: tissue Doppler imaging showing normal S wave velocity (0.11 m/s) and M-mode demonstrating normal TAPSE (1.9 cm); B) parasternal short-axis view showing normal pulmonary artery trunk diameter without evidence of thrombus; C) Doppler assessment of pulmonary pressures: pulmonary insufficiency flow; D) tricuspid regurgitation jet; E), and pulmonary artery acceleration time; F) demonstrating absence of pulmonary hypertension

**Figure 3:**
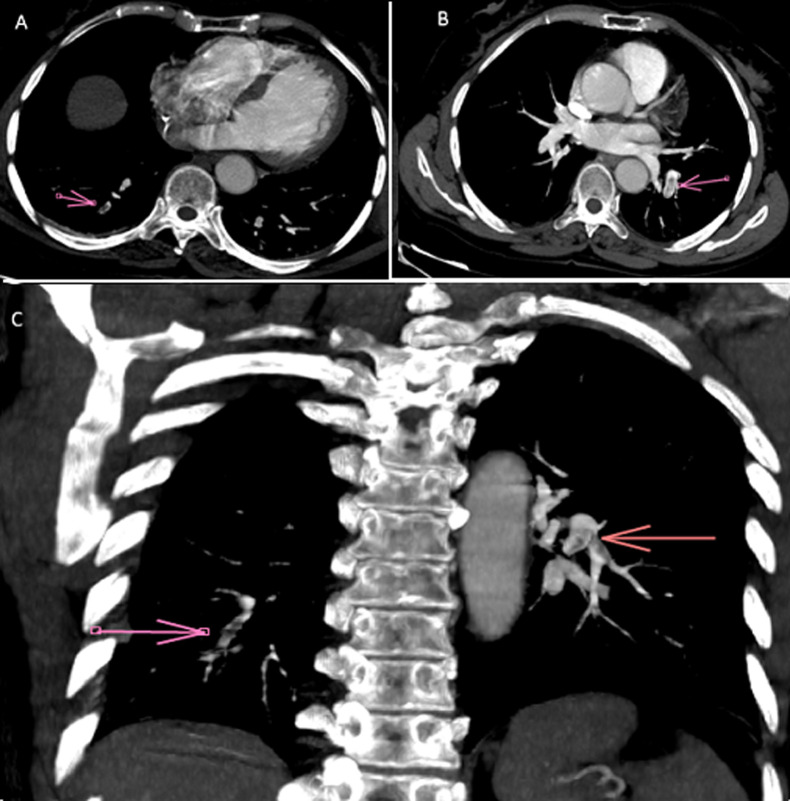
(A,B,C) computerized tomography pulmonary angiography images demonstrating segmental pulmonary emboli in the right posterobasal segment and the left lower lobe (arrows)

**Diagnostic assessment:** laboratory investigations on admission revealed a normal complete blood count with white blood cells at 6470 cells/mm^3^(normal 4000-10000/mm^3^), hemoglobin at 12.7 g/dL (normal for women 12-16 g/dl), and platelet count at 176000 cells /mm^3^(normal 150000-400000/mm^3^). Coagulation studies showed a prothrombin time of 99% with an activated partial thromboplastin time ratio of 1.00. Notably, fibrinogen was decreased at 1.8 g/L (normal 2-4 g/L). D-dimers were markedly elevated at 11.63 mg/L. Renal function was normal with creatinine at 4.95 mg/L (normal for women 5.07-11.1 mg/L) and urea at 0.32 g/L (normal 0.15-0.45 g/L). Liver enzymes were within normal limits: AST at 22 U/L (normal 5-34 U/L), ALT at 14 U/L (normal 0-55 U/L) with a normal serum lipase level of 12 UI/l (normal < 60U/l). Creatine kinase was mildly elevated at 256 U/L (normal for women 29-168 UI/l). C-reactive protein was normal at 3.93 mg/L (normal 0-5 mg/L). High-sensitivity troponin was within normal limits at 1.9 ng/L (<26 ng/l), and NT-proBNP was at 29.7 pg/mL. The electrocardiogram showed no abnormalities (Figure 1) and the chest X-ray was unremarkable with no infiltrates, pleural effusion, or cardiomegaly. Transthoracic echocardiography showed a non-dilated right ventricle with good systolic function. There was no paradoxical septal motion, right ventricular pressure overload, signs of pulmonary hypertension, or any other echocardiographic evidence of acute cor pulmonale. No visible thrombus was detected in the pulmonary artery. The left ventricle was non-dilated with good systolic function (ejection fraction of 60%) and left ventricular filling pressures were normal (Figure 2). Given the respiratory symptoms and markedly elevated D-dimers, a computed tomography pulmonary angiography (CTPA) was performed, which revealed segmental pulmonary embolism in the right posterobasal segment and left lower lobe without signs of right ventricular strain (Figure 3). Lower limb venous Doppler ultrasound did not reveal any deep vein thrombosis. An etiological workup was performed to rule out underlying malignancy. Cerebral CT angiography and cervico-thoraco-abdomino-pelvic contrast-enhanced CT scans showed no abnormalities, and tumor markers were negative. A formal thrombophilia screening was not performed, as this was a first episode of venous thromboembolism clearly linked to a temporary and identifiable risk factor.

**Diagnosis:** the diagnosis of pulmonary embolism at day 3 post-snake bite envenomation was established.

**Therapeutic interventions:** no antivenom was administered, as the patient presented beyond the optimal therapeutic window and had no life-threatening manifestations. The patient was immediately started on therapeutic anticoagulation with low molecular weight heparin (LMWH) at a curative dose. Supplemental oxygen was provided via nasal cannula. Supportive care included intravenous hydration and analgesics for local pain management. The clinical evolution on day 2 of hospitalization showed improvement in respiratory parameters. Laboratory tests demonstrated stabilization of fibrinogen at 1.7 g/L, with hemoglobin maintained at 12.6 g/dL and platelets at 164000 cells/mm^3^. Coagulation parameters remained stable, with prothrombin time of 99% and an activated partial thromboplastin time ratio of 1.00. The local edema began to regress progressively. After 3 days of LMWH, the patient was transitioned to oral anticoagulation with rivaroxaban 15 mg twice daily for 3 weeks, followed by 20 mg once daily for a total duration of 6 months. The patient no longer required supplemental oxygen and showed significant clinical improvement with resolution of dyspnea and progressive reduction of limb edema. No bleeding complications were observed during anticoagulation therapy.

**Follow-up and outcome:** the patient was discharged on day 6 of hospitalization in stable condition with oral anticoagulation and close outpatient follow-up. At 3-month follow-up, the patient remained asymptomatic with complete resolution of lower limb edema. Physical examination was unremarkable. Transthoracic echocardiography showed persistent normal left and right ventricular function. Anticoagulation was continued as planned for a total of 6 months, with excellent tolerance and favorable clinical evolution. The patient had no recurrence of thromboembolic events, and anticoagulation was successfully discontinued after 6 months with continued clinical surveillance

**Patient perspective:** the patient expressed optimism for a full recovery.

**Informed consent:** the patient provided written consent for the publication of his clinical details and any identifying images.

## Discussion

Annual snake bite incidence reaches 5 million cases globally, with envenomation documented in over 2 million victims and mortality reaching up to 125,000 deaths [1]. In Morocco, viperids account for 98% of 217 reported envenomations over three years (2001-2003) by the Rabat poison control center, with 6% mortality [2]. Although viper envenomation is relatively frequent, pulmonary embolism remains an exceptional complication. The typical presentation involves local tissue injury progressing from pain and swelling to necrosis or compartment syndrome, accompanied by systemic effects such as shock, acute renal failure, and coagulation disturbances [1]. Reported cases predominantly feature hemorrhagic manifestations resulting from consumption coagulopathy, whereas thrombotic events are far less common, including stroke (up to 6.4% incidence), coronary, or splanchnic ischemia [1]. Nonetheless, pulmonary embolism has occasionally been described following bites from several viper species, including Crotalus scutulatus, Moroccan vipers, Bothrops lanceolatus, and unidentified vipers in France and Greece [3]. Viper venom contains coagulation-modifying toxins including metalloproteinases, serine proteases, and C-type lectins that activate the pathological hemostatic cascade through 3 concomitant mechanisms: direct stimulation of platelet function, activation of coagulation factors II, V and X, and release of thrombin-mimicking proteases [1]. This massive coagulation activation creates unstable fibrin that degrades rapidly through fibrinolysis, depleting hemostatic reserves within 30 minutes and producing a hemorrhagic diathesis that defines the classic acute phase manifestation [4]. However, before complete fibrinolytic dissolution, microthrombi can migrate to distant organs, causing ischemic events [5]. Viper envenomation induces a biphasic hemostatic response: an acute consumptive phase with depletion of coagulation factors and thrombocytopenia, which may transition into a secondary prothrombotic phase characterized by reactive hyperfibrinogenemia and thrombocytosis [6].

Beyond this temporal biphasic evolution, certain venom components can produce a distinct pathophysiological phenomenon resembling disseminated intravascular coagulation without true disseminated intravascular coagulation (DIC) [7]. Specific thrombin-like venom proteases cleave both fibrinopeptides A and B from fibrinogen while activating factor XIII, generating cross-linked fibrin clots resistant to fibrinolysis [8]. This "pseudo-DIC" differs fundamentally from authentic DIC: unlike thrombin-mediated consumption of factors II and X seen in true DIC, venom-induced pseudo-DIC produces thrombotic potential through direct proteolytic activity without engaging the classical coagulation cascade [8]. Notably, these venom proteases are not inhibited by heparin, distinguishing them mechanistically from thrombin-dependent pathways [8]. Our patient presented at day 3 with normalized PT/aPTT and adequate platelet count, but markedly elevated D-dimers and mildly decreased fibrinogen. This biological profile, inconsistent with acute consumptive coagulopathy, suggested a hemostatic state conducive to thromboembolism. The absence of lower limb deep vein thrombosis despite pulmonary embolism represents a consistent finding across reported post-envenomation cases, suggesting mechanisms distinct from classical venous thromboembolism. Venom proteases may generate unstable fibrin polymers that preferentially aggregate in pulmonary vessels, or trigger direct in situ pulmonary thrombosis through systemic coagulation activation [7]. Alternatively, microthrombi from local tissue injury or thrombi in non-compressible venous segments may escape ultrasound detection.

Antivenom administration presented a challenging therapeutic decision. Although delayed antivenom administration generally shows reduced efficacy, it remains indicated when active envenomation is documented, with advanced age (>60 years) representing a criterion even in cases of minimal envenomation; in our patient, however, presentation on day 3 with normalized coagulation times and absence of active hemorrhage suggested that acute venom activity was limited [1,2]. The primary manifestation, pulmonary embolism, represented a thrombotic rather than toxic complication unlikely to respond to antivenom. Given the risks of hypersensitivity reactions and the uncertain benefit in reversing an already-established thromboembolic event, antivenom was not administered. Heparin is contraindicated during the acute phase as it does not neutralize venom proteases [9]. However, at day 3 with normalized hemostatic parameters, therapeutic anticoagulation was safely initiated. Despite extensive limb edema, fasciotomy was not performed, given preserved distal perfusion and absence of compartment syndrome. Pain control utilized opioid analgesics rather than nonsteroidal anti-inflammatory agents to minimize hemorrhagic risk [1]. Low-molecular-weight heparin achieved rapid clinical improvement, including resolution of respiratory distress, restoration of oxygen independence, and progressive regression of edema, without hemorrhagic complications, confirming that anticoagulation is safe once acute consumptive coagulopathy has resolved. This aligns with recommendations to initiate thromboprophylaxis after hemostatic correction, though we applied this therapeutically given documented thromboembolism [10]. The patient's favorable long-term outcome supports the appropriateness of our therapeutic approach. Six-month anticoagulation with rivaroxaban was completed without hemorrhagic complications. At three-month follow-up, complete clinical and echocardiographic resolution was documented. No thromboembolic recurrence occurred after anticoagulation discontinuation, validating the classification as provoked venous thromboembolism requiring finite-duration therapy

## Conclusion

This case illustrates that pulmonary embolism, though exceptionally rare, can complicate snake envenomation during the subacute phase. Thromboembolism may develop despite normalized coagulation parameters and absence of lower extremity deep vein thrombosis. Once coagulopathy resolves, therapeutic anticoagulation is safe and effective. This case underscores the importance of sustained monitoring for thromboembolic events following the acute phase of snake envenomation
